# The Great Pretender

**Published:** 1992-08

**Authors:** John Potokar

**Affiliations:** Registrar in Psychiatry, Susan Britten Wills Unit, The General Hospital, Bristol BS1 6SY


### The Great Pretender

**Dr. John Potokar, Mb.Ch.B., M.R.C.Psych.**
Registrar in Psychiatry,
Susan Britten Wills Unit,
The General Hospital, Bristol BS1 6SY

Although the incidence of neurosyphilis has considerably declined since the introduction of antibiotics, sporadic cases are still encountered. Atypical presentations occur and the classic grandiose picture may now be the exception. A recent example is described and the implications in clinical practice are discussed in the light of recent evidence, which suggests an association with the Acquired Immune Deficiency Syndrome (AIDS).

The discovery of Penicillin in 1941 led to a substantial improvement in the prognosis for neurosyphilis. Prior to this, there was usually a steady deteriation in the sufferers' condition. During the terminal stages of the disease, the patient would become incontinent, and ultimately lead a vegetative existence. Antibiotics, in particular Penicillin can often arrest the progress of the disease and hence it is essential to make the diagnosis as early as possible. However the use of antibiotics for other maladies may lead to partial treatment of syphilis during the earlier stages of the disease and this might be the explanation for the atypical presentations which can present.[1,2]

### CASE REPORT

Mr B first presented to an Accident and Emergency department in Bristol, having taken an overdose of fifty paracetamol tablets. He had became depressed over a period of six months, with biological features, including weight loss of two stones. There were no obvious precipitants. He was offered admission, but refused. Subsequently he was started on dothiepin and referred to outpatients. Further assessment revealed that he had been acting unusually prior to the overdose. His behaviour had become regressive during the preceeding three months. On occasions he would suck his thumb for long periods and when he spoke it was monosyllabically, in rather an infantile manner. At other times he had been getting on his hands and knees and acting like a dog.

It was noted then that he had suffered considerable stresses. His fourth wife was in the process of divorcing him, and he was in substantial debt. He had an extensive forensic history. Diagnoses that were considered were an hysterical reaction to life stresses, a schizophrenic illness and an atypical depression. He was prescribed thioridazine with some benefit and referred for psychometric testing. No clear picture emerged from this although it was noted that his lie scores were high and there was other evidence suggesting strong denial of any depressive symptoms. He deteriorated fairly rapidly over the next two months, taking to his bed, not washing nor shaving. He claimed not to be able to remember who he was. He was admitted to the psychiatric intensive care unit where he was found to be nihilistically deluded. He claimed that his brain was missing and that he had become a skeleton. At times he would growl like a dog, claiming to have changed species "to escape". He believed that his bowels did not exist, and he refused to accept that blood could be taken, saying "My veins have shrunk and withered away". He refused to eat and fluid intake was very poor. It was felt that he was suffering from a life threatening psychotic depression. He was therefore given a course of ECT with modest improvement, although he continued to have nihilistic delusions. He was transferred to the acute admission unit where review of his notes revealed that syphilis serology had not been obtained. Subsequent titres revealed a positive VDRL(1:128). IgG Elisa was positive. HIV was negative. On physical examination he had uniformly brisk reflexes with down going plantars. His right pupil was smaller than the left and unreactive to light, but not to accommodation (Argyll Robertson pupil). C.T scan demonstrated mild cerebral atrophy. A diagnosis of neurosyphilis was made and he was given a course of procaine penecillin 32 megaunits day, i.m.), with prednisolone cover. Although he remains in hospital three months after treatment his quality of life appears to have markedly improved. His speech is often spontaneous and much less nihilistic in content. He visits local shops and is far more independent than he was before treatment.

### DISCUSSION

In the case described the overwhelming picture was of a psychotic depression with nihilistic delusions. His repeated utterings of "my brain is missing" were not wholly inaccurate, in that CT scan demonstrated cerebral atrophy. Indeed the old adage "always listen to the patient" retrospectively took on an uncanny poignancy when his tortured utterings were further analysed. The constant repetition of " I'm a skeleton" became more significant. Although he did not appear malnourished he had lost approximately two stones in weight. With chemotherapy his weight returned to normal, although he continued to complain that he was a skeleton, albeit with less intensity. Depression is the most common psychiatric misdiagnosis,[3] although many other diagnoses may be given before the true culprit is elucidated eg schizophrenia, hypomania, dementia, epilepsy and acute confusional states. If the diagnosis is not to be missed, it is essential to consider syphilis in the aetiology of many psychiatric presentations.

This increased vigilance may be more relevant now with the evidence that the Human Immunodefficency virus may effect the natural history of syphilitic infection. Recent findings suggests that there are complex interactions between the two. Of particular importance are reports which suggest that patients with HIV infection who aquire syphilis are more likely to progress to clinical neurosyphilis, than those without HIV infection. This may be explained by the alteration in cell-mediated immunity that occurs with HIV infection.[4] A recent study from the United States found that 44% of patients with neurosyphilis had AIDs.[5] The commonest physical finding on clinical examination was an altered mental state, characteristically disorientation and memory loss.

Clearly the "Great Pretender" is still around and vigilance is called for if the diagnosis is not to be missed. This is especially true, in view of possible interactions with H.I.V., the "Young Pretender". It seems that Sir William Osler's advice is as true today, as it was when spoken nearly one hundred years ago: "Know syphilis in all its manifestations".

### ACKNOWLEDGEMENTS

With thanks to Drs. G. Bennet, D. Cook and J. Arumainayagam

### REFERENCES

(1) Burke J. M., Schaberg D. R. (1985). Neurosyphilis in the antibiotic era. *Neurology*. **35**, 1368-1371

(2) Hooshmand H., Escobar M. & Kopf S. (1972). Neurosyphilis: A study of 241 patients. *Journal of the American Medical Association*. Vol 219 No. 6 726-729.

(3) Dewhurst K. (1969). The neurosyphilitic psychoses today: A survey of 91 cases. *British Journal of Psychaitry*. **115**, 31-38.

(4) Johns D. R, Tierney M. & Felsenstein D. (1987). Alteration in the natural history of neurosyphilis by concurrent infection with the human immunodefficiency virus. *New England Journal of Medicine*. **316**, 1569-72.

(5) Katz D. & Berger J.(1989). Neurosyphilis in acquired immunodefficiency syndrome. *Archives of Neurology*. Vol. 46, 895-8.

